# Impact of the Absolute Difference in Diastolic Blood Pressure Between Arms in Patients With Coronary Artery Disease

**DOI:** 10.14740/jocmr2330w

**Published:** 2015-09-25

**Authors:** Yuka Hitaka, Shin-ichiro Miura, Rie Koyoshi, Yuhei Shiga, Yuiko Miyase, Kenji Norimatsu, Ayumi Nakamura, Sen Adachi, Takashi Kuwano, Makoto Sugihara, Amane Ike, Hiroaki Nishikawa, Keijiro Saku

**Affiliations:** a Department of Cardiology, Fukuoka University School of Medicine, Fukuoka 814-0180, Japan; b Department of Molecular Cardiovascular Therapeutics, Fukuoka University School of Medicine, Fukuoka 814-0180, Japan

**Keywords:** Diastolic blood pressure, Coronary artery disease, Difference in blood pressure between arms, Traditional risk factors

## Abstract

**Background:**

We investigated the relationship between the severity and presence of coronary artery disease (CAD) and a difference in systolic and diastolic blood pressure (SBP and DBP) between arms or between lower limbs.

**Methods:**

We enrolled 277 patients who underwent coronary angiography. We calculated the absolute (|right BP (rt. BP) - left BP (lt. BP)|) and relative (rt. BP - lt. BP) differences in SBP or DBP between arms or between lower limbs, and assessed the severity of CAD in terms of the Gensini score.

**Results:**

The absolute difference in DBP between arms in the CAD group was significantly lower than that in the non-CAD group, whereas the absolute difference in DBP between lower limbs in the CAD group was significantly higher. There were no differences in the absolute or relative difference in SBP between arms or lower limbs between the groups. The absolute difference in DBP between arms decreased as the Gensini score increased. In a logistic regression analysis, the presence of CAD was independently associated with the absolute difference in DBP between arms, in addition to male, family history, dyslipidemia, diabetes mellitus and hypertension.

**Conclusion:**

The absolute difference in DBP between arms in addition to traditional factors may be a critical risk factor for the presence of CAD.

## Introduction

According to the Japanese Society of Hypertension Guidelines for the Management of Hypertension 2014, bilateral brachial blood pressure (BP) should be measured at the initial clinic visit [1]. Generally, BP in the right arm is a few mm Hg higher than that in the left arm [2]. However, a difference in systolic BP (SBP) of more than 10 mm Hg between arms has been shown to be strongly associated with the presence of cardiovascular disease (CVD) and increased CV mortality [3-6]. We recently found that a relative difference in SBP between arms by synchronal measurement may be associated with the presence of coronary artery disease (CAD) [7]. Thus, a difference in BP between arms could be a useful indicator of the risk of CVD and mortality.

Since the difference in BP between arms could be a useful indicator of the risk of CVD, we hypothesized that a difference in BP could, along with traditional CV risk factors, predict the severity and presence of CAD. Therefore, we investigated whether the differences in SBP and DBP between arms and lower limbs were associated with the severity and presence of CAD.

## Methods

### Subjects

We enrolled 277 consecutive patients who were clinically suspected of having CAD, and who had at least one cardiac risk factor or an abnormality in their electrocardiogram, such as ST depression, negative T wave, or left bundle branch block. All patients underwent coronary computed tomography angiography (CTA) or invasive coronary angiography (CAG) and an assessment of brachial-ankle pulse wave velocity (baPWV). We excluded patients with unstable angina or myocardial infarction within the previous 4 weeks, chronic renal disease with hemodialysis or peripheral artery disease. We divided the patients into two groups, a non-CAD group (n = 86) and a CAD group (n = 191), and defined the severity of coronary atherosclerosis according to the Gensini score. Stable angina (CAD group) was defined as no changes in the frequency, duration, or intensity of symptoms for 4 weeks and as lumen diameter stenosis > 50% by CAG or CTA in at least one major coronary artery. The protocol in this study was approved by the Ethics Committee of Fukuoka University Hospital. We retrospectively collected and analyzed all data using the database of Fukuoka University Hospital.

### Assessment of CV risk factors

Data on weight, height, serum levels of triglycerides (TG), high-density lipoprotein cholesterol (HDL-C), and low-density lipoprotein cholesterol (LDL-C), estimated glomerular filtration rate (eGFR), uric acid (UA) and hemoglobin A1c (HbA1c) were collected. The patient characteristics, including the history of hypertension (HTN), dyslipidemia (DL), diabetes mellitus (DM), smoking status (current versus non-smokers), family history (myocardial infarction, angina pectoris, or sudden death), and medication use were obtained from medical records. Patients who had a current SBP/DBP ≥ 140/90 mm Hg or who were receiving antihypertensive therapy were considered to have HTN. Patients with LDL-C ≥ 140 mg/dL and/or TG ≥ 150 mg/dL or HDL-C ≤ 40 mg/dL, or who were receiving lipid-lowering therapy were defined as DL. DM was defined using the Japanese Diabetes Society criteria. Body mass index (BMI) was calculated as weight (kg)/height^2^ (m^2^).

### Measurement of baPWV

BaPWV was measured with subjects in the supine position using a volume-plethysmographic device (PWV/ABI, OMRON COLIN Co., Ltd, Tokyo, Japan) as described previously [8]. The following equation was used to obtain baPWV: baPWV = (La (path length from the suprasternal notch to the ankle) - Lb (path length from the suprasternal notch to the brachium))/ΔTba (time interval between the brachium and ankle). In all studies, baPWV was obtained after at least 5 min of rest. Mean PWV (mPWV) was calculated as (right (rt.) baPWV + left (lt.) baPWV)/2. We measured BP in the arms and lower limbs using a volume-plethysmographic device and calculated the absolute (|rt. BP - lt. BP|) and relative (rt. BP - lt. BP) differences in SBP and DBP between arms.

### Statistical analysis

Statistical analysis was performed using SAS9.3 (SAS Institute Inc.). Data are shown as the mean ± standard deviation (SD). Categorical and continuous variables were compared between the groups by a Chi-square analysis and unpaired or paired *t*-test, respectively. Multivariate analysis was performed using a logistic regression analysis for independent variables among traditional CV risk factors that were related to the absolute differences in DBP between arms and between lower limbs. A value of P < 0.05 was considered significant.

## Results

### Patient characteristics in all patients, and in the non-CAD and CAD groups

Table 1 shows the characteristics in all of the patients, and in the non-CAD and CAD groups. The average age, % male and BMI in all patients were 66.5 years, 70% and 23.7 kg/m^2^, respectively. There were many significant differences between the non-CAD and CAD groups, including gender, smoking, DL, HTN, DM, and medications including angiotensin II receptor blocker (ARB)/angiotensin converting enzyme inhibitor (ACE-I), calcium channel blocker (CCB), β-blockers, and statin. The % smokers, DL, HTN and DM in the CAD group were significantly higher than those in the non-CAD group. With regard to the use of medications, % of ARB/ACE-I, CCB, β-blockers and statin in the CAD group were significantly higher than those in the non-CAD group.

**Table 1:** Patient Characteristics in All Patients, Non-CAD and CAD Groups

	All (n = 277)	Non-CAD (n = 86)	CAD (n = 191)
Age, years	66.5 ± 9.8	65.2 ± 10.8	67.1 ± 9.3
Male, n (%)	193 (70)	47 (55)	146 (76)*
BMI, kg/m^2^	23.7 ± 3.3	23.3 ± 3.3	23.9 ± 3.3
Family history, n (%)	63 (23)	13 (15)	50 (26)*
Smoking, n (%)	146 (53)	37 (43)	109 (57)*
HTN, n (%)	213 (77)	53 (62)	160 (84)*
DL, n (%)	232 (84)	58(67)	174 (91)*
HDL-C, mg/dL	52 ± 13	58 ± 13	50 ± 12*
LDL-C, mg/dL	103 ± 30	115 ± 29	97 ± 29*
TG, mg/dL	132 ± 74	124 ± 61	136±79
DM, n (%)	107 (39)	17 (20)	90 (47)*
HbA1c, %	6.1 ± 1.1	5.8±0.8	6.2 ± 1.2*
HU, n (%)	50 (18)	15(17)	35 (18)
UA, mg/dL	5.4 ± 1.3	5.3 ± 1.2	5.5 ± 1.3
eGFR, mL/min/1.73 cm^2^	64 ± 16	66 ± 14	63 ± 16
baPWV (mean), cm/s	1,734 ± 368	1,694 ± 315	1,753 ± 389
Medication, n (%)			
ARB/ACE-I	177 (64)	14 (16)	47 (25)*
CCB	134 (48)	31 (36)	103 (54)*
α-blocker	3 (1)	0	3 (2)
β-blocker	43 (16)	8 (9)	35 (18)*
Diuretics	40 (14)	10 (12)	30 (16)
Statin	192 (69)	32 (37)	160 (84)*
Ezemitibe	22 (8)	3 (3)	19 (10)
Insulin	15 (5)	2 (2)	13 (7)
Sulfonyurea	34 (12)	-11	25 (13)
Pioglitazone	6 (2)	1 (1)	5 (3)

BMI: body mass index; HTN: hypertension; DL: dyslipidemia; HDL-C: high-density lipoprotein cholesterol; LDL-C: low-density lipoprotein cholesterol; TG: triglyceride; DM: diabetes mellitus; HbA1c: hemoglobin A1c; HU: hyperurecemia; UA: uric acid; eGFR: estimated glomerular filtration rate; baPWV: brachial-ankle pulse wave velocity; ARB/ACE-I: angiotensin II receptor blocker/angiotensin converting enzyme inhibitor; CCB: calcium channel blocker. *P < 0.05 vs. non-CAD group.

### Evaluation of baPWV and SBP/DBP in the rt. and lt. arms and lower limbs

Table 2 shows the baPWV, and SBP/DBP in the rt. and lt. arms and lower limbs in all of the patients, and in the non-CAD and CAD groups. There was no difference in baPWV between the non-CAD and CAD groups. The rt. and lt. SBP/DBP in all patients were 130.4/76.1 and 132.8/78.4 mm Hg, respectively. The absolute difference in DBP between arms in the CAD group was significantly lower than that in the non-CAD group, whereas the absolute difference in DBP between lower limbs in the CAD group was significantly higher. There were no differences in the absolute or relative difference in SBP between arms or lower limbs between the CAD and non-CAD groups.

**Table 2:** Rt. and Lt. BP, Absolute and Relative BP Differences Between Arms and Lower Limbs in All Patients, Non-CAD and CAD Groups

	All (n = 277)	Non-CAD (n = 86)	CAD (n = 191)
Rt. SBP arm, mm Hg	130.4 ± 18.0	132.8 ± 19.3	129.3 ± 17.4
Lt. SBP arm, mm Hg	129.9 ± 18.0	132.5 ± 18.7	128.7 ± 17.6
M. SBP arm, mm Hg	130.1 ± 17.8	132.6 ± 18.7	129.0 ± 17.3
Rt. DBP arm, mm Hg	76.1 ± 10.4	78.4 ± 11.0	75.0 ± 9.9*
Lt. DBP arm, mm Hg	75.8 ± 10.1	78.1 ± 10.6	74.8 ± 9.8*
M. DBP arm, mm Hg	75.9 ± 10.1	78.2 ± 10.6	74.9 ± 9.7*
Rt. SBP lower limb, mm Hg	147.3 ± 26.7	152.5 ± 23.2	144.9 ± 27.9*
Lt. SBP lower limb, mm Hg	145.5 ± 24.7	151.1 ± 22.1	143.0 ± 25.5*
M. SBP lower limb, mm Hg	146.4 ± 24.9	151.8 ± 22.2	144.0 ± 25.8*
Rt. DBP lower limb, mm Hg	74.0 ± 10.7	77.0 ± 10.5	72.6 ± 10.5*
Lt. DBP lower limb, mm Hg	74.7 ± 10.7	77.1 ± 10.6	73.6 ± 10.6*
M. DBP lower limb, mm Hg	74.3 ± 10.4	77.1 ± 10.4	73.1 ± 10.2*
Absolute Dif. SBP arms, mm Hg	3.2 ± 3.1	3.0 ± 2.6	3.3 ± 3.4
Relative Dif. SBP arms, mm Hg	0.7 ± 4.4	0.8 ± 3.9	0.6 ± 4.6
Absolute Dif. SBP limbs, mm Hg	7.5 ± 8.6	6.6 ± 6.1	7.9 ± 9.5
Relative Dif. SBP limbs, mm Hg	2.3 ± 11.2	1.6 ± 8.9	2.4 ± 12.2
Absolute Dif. DBP arms, mm Hg	3.1 ± 2.7	3.7 ± 3.0	2.7 ± 2.6*
Relative Dif. DBP arms, mm Hg	0.2 ± 4.1	0.1 ± 4.8	0.2 ± 3.7
Absolute Dif. DBP limbs, mm Hg	3.4 ± 3.3	2.8 ± 2.6	3.7 ± 3.5*
Relative Dif. DBP limbs, mm Hg	3.2 ± 3.1	3.0 ± 2.6	3.3 ± 3.4
SBP absolute arms ≥ 10 mm Hg, n (%)	12 (4)	2 (20)	10 (5)
DBP absolute arms ≥ 10 mm Hg, n (%)	10 (4)	5 (16)	5 (3)
SBP absolute lower limbs ≥ 10 mm Hg, n (%)	69 (24)	17 (19)	52 (27)
DBP absolute lower limbs ≥ 10 mm Hg, n (%)	1 (6)	4 (5)	12 (6)

Rt. SBP: right systolic blood pressure; Lt. SBP: left SBP; M. SBP: mean SBP; Rt. DBP: right diastolic BP; Lt. DBP: left DBP; M. DBP: mean DBP; Dif. BP arms: differences in BP between arms; Dif. BP limbs: differences in BP between lower limbs. *P < 0.05 vs. non-CAD group.

### Associations between baPWV, the absolute differences in DBP between arms or lower limbs and various parameters

BaPWV was significantly associated with age, BMI, SBP/DBP in the arms and lower limbs, HbA1c and eGFR (Table 3). Although the absolute difference in DBP between lower limbs was significantly associated with HDL-C and eGFR, these associations were very weak. In addition, there was no association between the absolute difference in DBP between arms and between lower limbs.

**Table 3:** Associations Between baPWV, Absolute Differences in DBP Between Arms, and Other Various Parameters

	baPWV (mean)		Absolute Dif. DBP arms		Absolute Dif. DBP limbs	
	r	P value	r	P value	r	P value
Age, years	0.52	< 0.0001	0.03	0.68	0.11	0.08
BMI, kg/m^2^	-0.13	0.03	0.11	0.08	-0.07	0.26
M. SBP arm, mm Hg	0.36	< 0.0001	0.11	0.06	0.10	0.10
M. DBP arm, mm Hg	0.12	0.04	0.09	0.15	0.02	0.72
M. SBP lower limb, mm Hg	0.42	< 0.0001	0.04	0.46	-0.02	0.78
M. DBP lower limb, mm Hg	0.27	< 0.0001	0.05	0.44	-0.12	0.04
LDL-C, mg/dL	-0.01	0.77	0.06	0.34	-0.12	0.05
HDL-C, mg/dL	-0.01	0.86	0.01	0.81	-0.13	0.04
TG, mg/dL	-0.05	0.38	-0.02	0.73	-0.06	0.31
HbA1c, %	0.14	0.02	-0.11	0.06	0.01	0.83
eGFR, mL/min/1.73 cm^2^	-0.26	< 0.0001	0.02	0.80	-0.16	0.01
UA, mg/dL	-0.07	0.26	-0.0003	0.99	0.04	0.48
baPWV (mean), cm/s	-	-	-0.04	0.50	0.05	0.41
Absolute Dif. DBP arms, mm Hg	-0.04	0.50	-	-	0.07	0.28
Absolute Dif. DBP limbs, mm Hg	0.05	0.41	0.07	0.28	-	-

BMI: body mass index; M. SBP: mean systolic blood pressure; M. DBP: mean diastolic BP; LDL-C: low-density lipoprotein cholesterol; HDL-C: high-density lipoprotein cholesterol; TG: triglyceride; HbA1c: hemoglobin A1c; eGFR: estimated glomerular filtration rate; UA: uric acid; baPWV: brachial-ankle pulse wave velocity; Dif. BP arms: differences in BP between arms; Dif. BP limbs: differences in BP between lower limbs. *P < 0.05 vs. non-CAD group.

### Associations between the absolute difference in DBP between arms or lower limbs and the Gensini score

**Figure 1:**
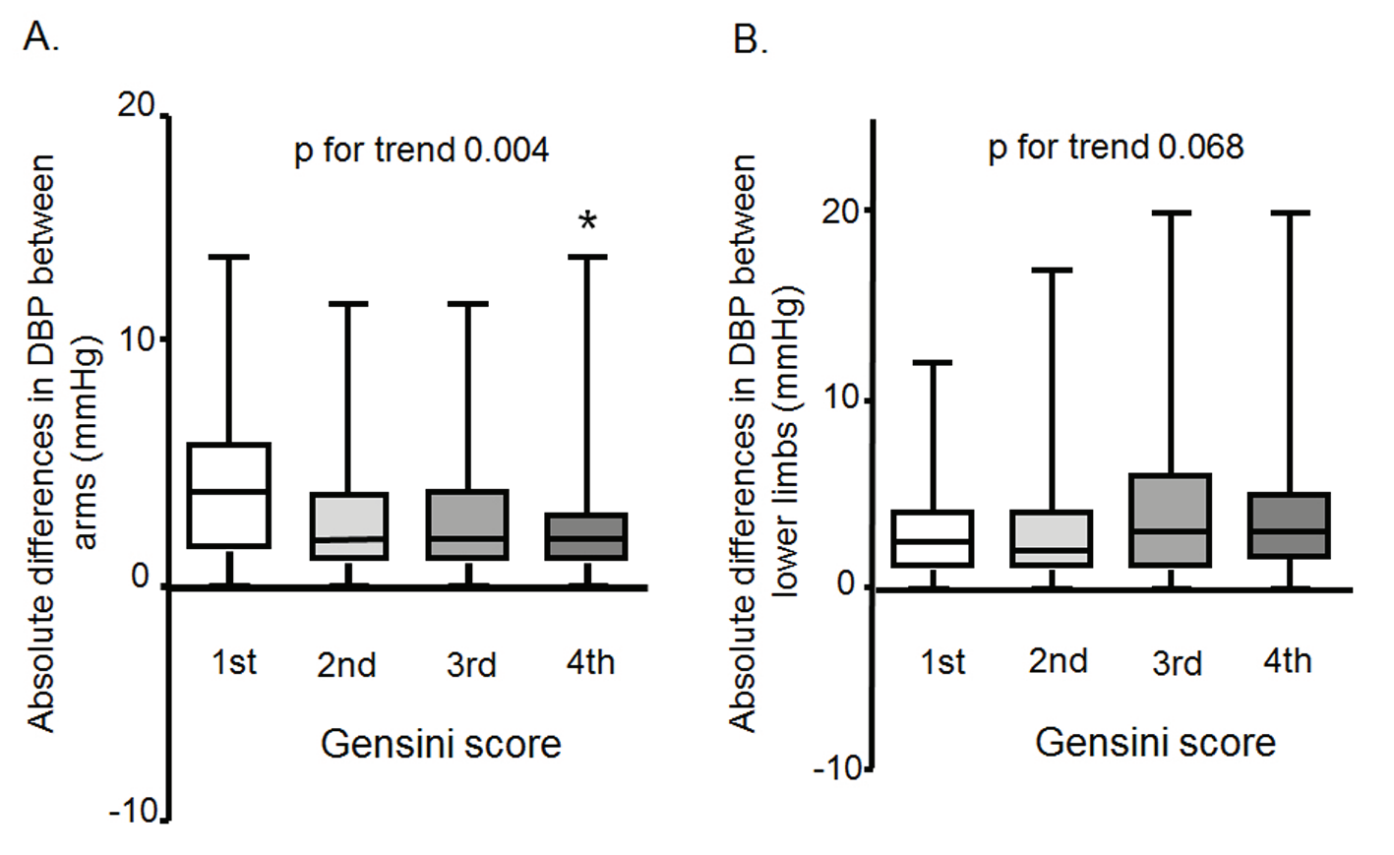
Associations between the absolute difference in DBP between arms (A) or lower limbs (B) and the Gensini score.

We divided the patients into quartiles according to the Gensini score (Fig. 1). The absolute difference in DBP between arms decreased as the Gensini score increased, as shown in Figure 1A. There was no association between the absolute difference in DBP between lower limbs and the Gensini score (Fig. 1B).

### Predictors of the presence of CAD

**Figure 2:**
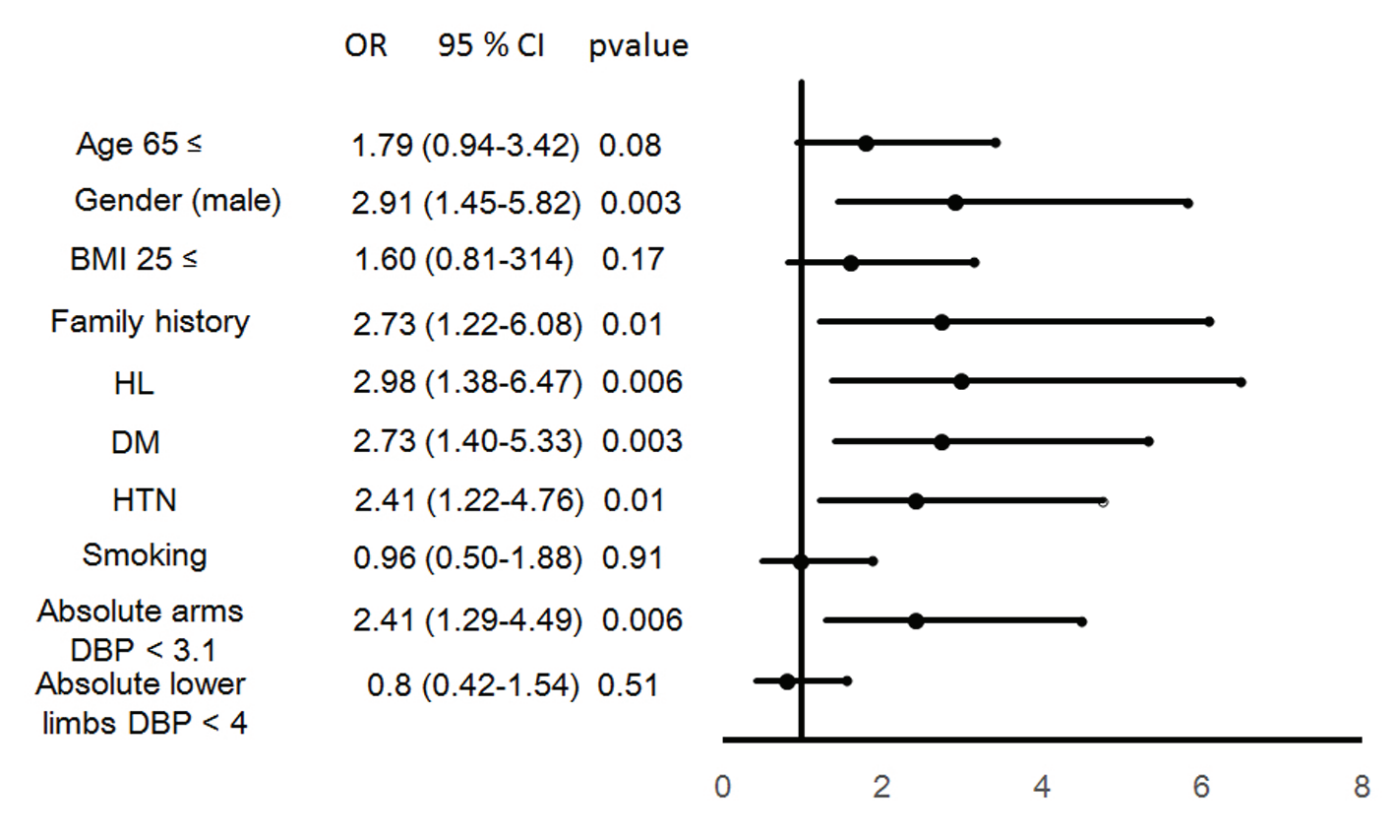
Predictors of the presence of CAD.

To confirm the parameters that predicted the presence of CAD, we performed a logistic regression analysis using independent variables (absolute differences in DBP between arms < 3.1 mm Hg (mean values in all patients) and lower limbs < 4.0 mm Hg (mean values in all patients) in addition to traditional coronary risk factors (age ≥ 65 years, gender male, BMI ≥ 25 kg/m^2^, DL, DM, HTN, family history and smoking)) that were related to CAD (Fig. 2). CAD was independently associated with the absolute difference in DBP between arms in addition to male, family history, DL, DM and HTN.

## Discussion

In this cross-sectional study, we assessed the interrelationship between the absolute and relative differences in BP between arms or lower limbs and the presence of CAD. First, we found that the absolute difference in DBP between arms in patients with CAD was significantly lower than that in patients without CAD, whereas the absolute difference in DBP between lower limbs in CAD group was significantly higher. Second, the absolute difference in DBP between arms decreased as the Gensini score increased. Finally, among the absolute difference in DBP between arms or lower limbs and traditional CV risk factors (age, male, BMI, smoking, family history, DM, DL and HTN), the absolute difference in DBP between arms, male, family history, HTN, DL and DM were independently associated with the presence of CAD by a logistic regression analysis.

The most interesting finding was that the absolute difference in DBP between arms, but not the absolute difference in SBP between arms, was associated with the severity and presence of CVD. Previous reports have mainly indicated that the difference in SBP, but not DBP, was associated with the severity and presence of CAD and increased CV mortality [3-7]. An absolute difference in BP between arms was associated with CV risk factors in a general population in Japan [9]. In this study, the absolute difference in SBP between arms was not associated with the severity or presence of CAD. However, traditional CV risk factors (male, family history, HTN, DL and DM) were independent predictors of the presence of CAD. These results indicated that the patients in this study did not represent any specific patient group with CAD. In addition, Clark et al found that 8.6% of participants with diabetes and 2.9% of controls had systolic interarm differences ≥ 10 mm Hg [10]. Patients with CAD (19.4%) had a significantly higher prevalence of an absolute BP difference ≥ 10 mm Hg than non-CAD patients (2.7%) [11]. In this study, the absolute difference in SBP was about 3.2 mm Hg, and only 4% of all patients had an absolute SBP difference ≥ 10 mm Hg. Although we do not know why the absolute difference in SBP was not important for predicting the presence of CAD in this study, a lower prevalence of an absolute BP difference ≥ 10 mm Hg may have contributed to this finding. A few reports have stated that the difference in DBP between arms is associated with CV events [3-7]. Among these reports, only Clark et al stated that the difference in DBP between arms was associated with an increased hazard of CV events and combined non-fatal events or all-cause mortality, albeit with less precision than the difference in SBP between arms. In addition, in this study, a smaller, but not larger, absolute difference in DBP between arms, was associated with the severity and presence of CAD. The population-based Framingham Heart Study revealed that the CAD risk increased with a lower DBP at any level of SBP ≥ 120 mm Hg in the middle-aged and elderly [12]. CV complications may be increased with a fall in BP, especially DBP (J-curve phenomenon between BP and CAD) [13]. Since the coronary arteries are perfused predominantly during diastole, a J-curve, if any, should be most apparent for DBP and CV events. In the CAD group, mean SBP and DBP were 129.0 and 74.9 mm Hg, respectively (Table 2). Since DBP in the CAD group was significantly lower than that in the non-CAD group, a lower DBP with a smaller absolute difference in DBP between arms might influence increases in the presence of CAD. Nonetheless, there was no significant association between mean DBP in arms and the absolute difference in DBP between arms in this study (r = 0.09, P = 0.15) (Table 3). Moreover, it is widely known that BP is lower in the arm on the side with advanced arteriosclerosis (or atherosclerosis), and such an interarm difference significantly increases as arteriosclerosis advances. This theory also was not consistent with our results. Although this is the first report to show the relationship between a difference in DBP between arms and the severity and presence of CAD, we do not know why a smaller absolute difference in DBP was important for predicting them at this time. Further studies will be needed to clarify this issue.

In a logistic regression analysis, the presence of CAD was independently associated with traditional CV risk factors (male, family history, HTN, DL and DM). Differences in BP between the left and right arms have also been observed in patients with HTN [14] and DM [15]. Although there were no differences in BP between arms between the absence and presence of HTN or DM (data not shown), this may affect the absolute difference in DBP.

### Study limitations

This study has several limitations. First, the study was cross-sectional and included a relatively small number of patients. Second, bilateral BP measurements were performed after various treatments. Many of the patients were taking anti-hypertensive, anti-dyslipidemic and/or anti-diabetic medications that may have influenced the measurements of the differences in BP between arms. It is reasonable to expect that the patients with CAD were taking more medications than non-CAD patients. There were no significant differences in the absolute difference in BP between arms between patients with and without various medications. Prospective studies will be needed to clarify these limitations.

### Conclusion

The absolute difference in DBP between arms in addition to traditional factors may be a critical risk factor for the presence of CAD.
